# A Genome-Wide Survey of the Microsatellite Content of the Globe Artichoke Genome and the Development of a Web-Based Database

**DOI:** 10.1371/journal.pone.0162841

**Published:** 2016-09-20

**Authors:** Ezio Portis, Flavio Portis, Luisa Valente, Andrea Moglia, Lorenzo Barchi, Sergio Lanteri, Alberto Acquadro

**Affiliations:** 1 Dipartimento di Scienze Agrarie, Forestali ed Alimentari (DISAFA) - Plant Genetics and Breeding, University of Torino, I-10095 Grugliasco, Torino, Italy; 2 Yebokey – User Experience Design and Web Development, I-10134, Torino, Italy; University of Perugia, ITALY

## Abstract

The recently acquired genome sequence of globe artichoke (*Cynara cardunculus* var. *scolymus*) has been used to catalog the genome’s content of simple sequence repeat (SSR) markers. More than 177,000 perfect SSRs were revealed, equivalent to an overall density across the genome of 244.5 SSRs/Mbp, but some 224,000 imperfect SSRs were also identified. About 21% of these SSRs were complex (two stretches of repeats separated by <100 nt). Some 73% of the SSRs were composed of dinucleotide motifs. The SSRs were categorized for the numbers of repeats present, their overall length and were allocated to their linkage group. A total of 4,761 perfect and 6,583 imperfect SSRs were present in 3,781 genes (14.11% of the total), corresponding to an overall density across the gene space of 32,5 and 44,9 SSRs/Mbp for perfect and imperfect motifs, respectively. A putative function has been assigned, using the gene ontology approach, to the set of genes harboring at least one SSR. The same search parameters were applied to reveal the SSR content of 14 other plant species for which genome sequence is available. Certain species-specific SSR motifs were identified, along with a hexa-nucleotide motif shared only with the other two Compositae species (sunflower (*Helianthus annuus*) and horseweed (*Conyza canadensis*)) included in the study. Finally, a database, called “*Cynara cardunculus* MicroSatellite DataBase” (*CyMSatDB*) was developed to provide a searchable interface to the SSR data. *CyMSatDB* facilitates the retrieval of SSR markers, as well as suggested forward and reverse primers, on the basis of genomic location, genomic *vs* genic context, perfect *vs* imperfect repeat, motif type, motif sequence and repeat number. The SSR markers were validated via an *in silico* based PCR analysis adopting two available assembled transcriptomes, derived from contrasting globe artichoke accessions, as templates.

## Introduction

The allogamous Asteraceae species *Cynara cardunculus* L. (2n = 2x = 34) is native to the Mediterranean Basin and includes the three fully cross-compatible taxa designated as the globe artichoke (var. *scolymus*), the cultivated cardoon (var. *altilis*) and the wild cardoon (var. *sylvestris*); the latter is assumed to be the ancestor of the former two cultivated forms [1, 2]. Globe artichoke is grown for its edible immature inflorescences, while cultivated cardoon is cultivated mainly for its edible fleshy stems. Most of the world's production of globe artichoke is centered around the Mediterranean Basin, especially Italy [3]. The species was probably domesticated in Italy, where the most diverse primary genepool remains. At present, over 100 varieties are cultivated: they are generally classified on the basis of harvest time (early *vs* late) and/or inflorescence type (size, shape, presence/absence of spines, pigmentation of the outer bracts). While seed-propagated varieties and F_1_ hybrids are growing in popularity, vegetatively propagated planting material is still widely used, particularly by producers of local varietal types.

Advances in DNA sequencing technology have greatly simplified the acquisition of medium-sized and even large genomes. So far, almost 100 plant genome sequences have been completed, and the genomes of a further 400 are at various stages of completion (genomevolution.org, 6/2016). The globe artichoke genome sequence has recently been assembled into some 13,000 scaffolds, which have been assigned to the 17 chromosomes [4]. Whole genome sequences provide a wealth of genetic assays, exploitable for the assessment of genetic diversity, for genetic analysis, for gene isolation and for marker-assisted breeding. Simple sequence repeats (SSRs), which are ubiquitous and highly polymorphic are particularly favored in a plant breeding context, largely because they are usually co-dominantly inherited and can be readily assayed by conventional PCR. Although single nucleotide polymorphism markers are even more ubiquitous, SSRs have the advantage of being potentially multi-allelic [5]. Besides, the globe artichoke genome sequence has been recently assembled into 13K scaffolds (N50 = 125 Kbp, L50 = 1411), and organized in 17 reconstructed pseudomolecules [4], which were structurally and functionally annotated. The aim of the current study was to survey the globe artichoke genome for its SSR content. In so doing, it has been possible to construct a public domain database (http://www.artichokegenome.unito.it/cymsatdb/) of utility to the global community of globe artichoke scientists/breeders and, by exploiting the potential transferability to related taxa of many microsatellite loci, useful for a wider community of researchers studying Asteraceae.

## Materials and Methods

### The SSR content of the globe artichoke genome

The globe artichoke genome sequence recently reported by Scaglione *et al*. [4], and available in the public domain (PRJNA238069), was downloaded in FASTA format. Its 17 pseudomolecules (representing partial sequences of each of the species' chromosomes), along with all unmapped scaffolds, were chopped into manageable pieces using SciRoKo tool (http://kofler.or.at/bioinformatics/SciRoKo). Perfect, compound and imperfect SSRs were identified *in silico* using the SciRoKo SSR—search module (kofler.or.at/bioinformatics/SciRoKo). The thresholds applied were four repeats and a length of 15 nt. Thus, all declared perfect SSRs comprised a minimum of 15 mononucleotides, eight dinucleotides, five trinucleotides, four tetranucleotides, three pentanucleotides or three hexanucleotides. For compound repeats, the maximum spacer length was set at 100 nt. The coordinates (start/end position) of each SSR were matched with those coordinates of the gene space using Bedtools intersect (http://bedtools.readthedocs.io), using the default parameters with -loj (left outer join) option: where the overlap comprised at least 1 nt, the repeat was designated as a genic SSR. The putative function of genes harboring at least one SSR was determined by the GO (gene ontology) approach. Enrichment for GO terms was identified by comparing the set of genic SSRs against the whole genome GO annotation dataset using the AgriGO program (bioinfo.cau.edu.cn/agriGO/), collecting enriched GO terms with P values <e−5 and a false discovery rate <0.01, and visualized using ReviGO (revigo.irb.hr/).

### *CyMSatDB*, an SSR database for globe artichoke

The *CyMSatDB* (*Cynara cardunculus* MicroSatellite DataBase) was developed to provide browsable access to the SSR data. This web application, based on a LAMP stack, comprises a client tier (client browser), a middle tier (Apache web server with PHP interpreter) and a database tier (MySQL DBMS). A user-friendly interface was developed using PHP, which is an open-source server-side scripting language. The set of *in silico* detected SSRs was stored in the MySQL database, using PHP scripts to parse the text file from SciRoKo. User need-based customized queries are generated from the web interface and allow users to search the microsatellite marker information in MySQL database. A stand-alone version of Primer3 (https://sourceforge.net/projects/primer3) is provided to design primer pairs for any given SSR: its output lists alternative sets of primer pairs, and the characteristics of the expected amplicon. The validation screening was attempted using isPCR script (genome.ucsc.edu/) applying default parameters. Validated positive markers were counted and multiple PCR products were filtered for redundancy using a custom bash script.

### Collection of genomic sequences from different sources

For comparison purposes, the full genome sequences of 14 other plant species (sunflower [*Helianthus annuus*], horseweed [*Conyza canadensis*], tomato [*Solanum lycopersicum*], *Arabidopsis thaliana*, cotton [*Gossypium arboretum*], sweet orange [*Citrus sinensis*], apple [*Malus domestica*], cucumber [*Cucumis sativus*], common bean [*Phaseolus vulgaris*], black cottonwood [*Populus trichocarpa*], grapevine [*Vitis vinifera*], rice [*Oryza sativa*], date palm [*Phoenix dactilifera*] and banana [*Musa acuminata*]) were collected from the related public database and scanned for the presence of perfect SSRs using the same procedures described above. The source of each of the above full genome sequences is given in S1 Table.

## Results

### The SSR content of the globe artichoke genome

The ~725 Mbp of globe artichoke genome sequence yielded 177,207 perfect SSRs, equivalent to an overall density across the genome of 244.5 SSRs/Mbp; of these about 21% (37,748) were compound loci. The cumulative length of the full collection of SSRs was ~4.4 Mbp, or about 0.6% of the assembled genome (Table 1). The most common motifs (~73% of all SSRs, density of 178.6 SSRs/Mbp) were dinucleotides, followed by the tri- (~11%), the tetra- (~6%) and the mononucleotides (~5%). Both penta- and hexanucleotide repeats were uncommon (together < 5%) (Table 1). The total length of dinucleotide sequences was much larger than the other repetitive sequences, with a total of 3.3 Mbp (74.7% of the cumulative length of all SSR motifs). More than 224,000 imperfect SSRs were detected. Among this group, the occurrence of mono-, di-, tri- and tetranucleotide motifs was quite similar to that seen among the group of perfect SSRs, but that of the larger motifs was rather higher: together the penta- and hexanucleotide SSRs represented ~18% of the set of imperfect SSRs (Table 1).

**Table 1:** Variation in repeat length among genomic globe artichoke perfect and imperfect SSRs.

SSR Type	Perfect motif							Imperfect motif		
	Kinds	Count	%	Density (SSRs/Mbp)	Cumulative (Mbp)	Cumulative (%)	Mean repeat number	Count	%	Density (SSRs/Mbp)
Mono-	2	8,393	4.7%	11.6	0.15	3.4%	17.7	8,716	3.9%	12.0
Di-	4	12,9390	73.0%	178.6	3.30	74.7%	12.7	13,7758	61.5%	190.1
Tri-	10	19,909	11.2%	27.5	0.44	10.0%	7.4	22,121	9.9%	30.5
Tetra-	33	10,742	6.1%	14.8	0.27	6.1%	6.3	15,485	6.9%	21.4
Penta-	95	4,302	2.4%	5.9	0.10	2.3%	4.8	23,992	10.7%	33.1
Hexa-	284	4,471	2.5%	6.2	0.15	3.5%	5.8	15,990	7.1%	22.1
**Total/mean**	**428**	**177,207**	**100.0%**	**244.5**	**4.41**	**100.0%**	**11.61**	**224,062**	**100.0%**	**309.2**

### Variation in SSR length, repeat number and motif

**Fig 1:**
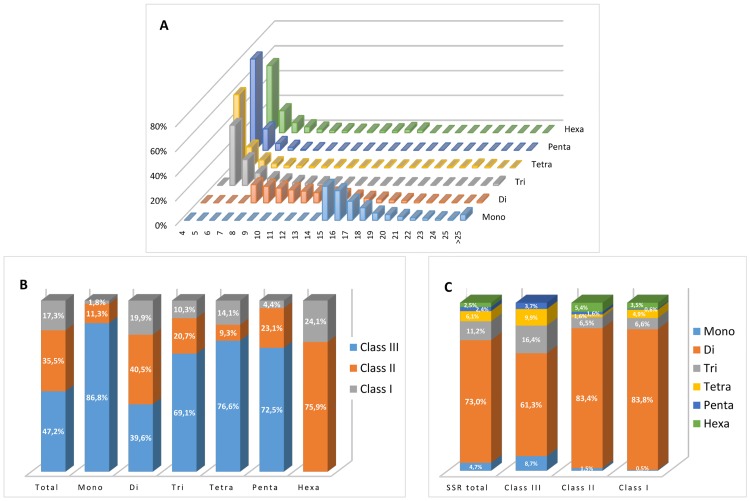
Perfect SSRs in the globe artichoke genome. (A) The frequency of repeat classes (class I >30 nt, class II 20–30 nt, class III <20 nt. (B) The distribution of motif type within each class. (C) The relative frequency of motifs.

The distribution of repeat unit number among the set of perfect SSRs is summarized in Fig 1, and given in full in S2 Table. For all SSR classes, the frequency was inversely proportional to the number of repeat units; for example, SSRs composed of ten or fewer repeat accounted for 49.2% of the set, while those harboring >20 repeats represented <5% of the set. The decline in frequency with greater length was least well marked for the mono- and dinucleotide SSRs, and most marked for the pentanucleotide SSRs (Fig 1a). As a result, the mean number of repeat units found among the set of dinucleotide SSRs (12.7) was nearly double that of in either the tri- or the tetranucleotide SSRs (7.4 and 6.3 respectively) and more than double that in the penta- and hexanucleotide ones (4.8 and 5.8, respectively) (Table 1). With respect to overall length, 17.3% of the SSRs were classified as long and hypervariable class I class I (>30 nt), 35.5% as potentially variable class II (20–30 nt) and the remaining 47.2% as variable class III (<20 nt) (Fig 1b). A preponderance (86.8%) of the mononucleotide SSRs belonged to class III, while 24.1% of the hexanucleotide SSRs were assigned to class I. The most variable class I loci harbored dinucleotides (83.8%), followed by those harboring tri- (6.6%), tetra- (4.9%) and hexanucleotides (3.5%); while mono- and penta-nucleotide were almost absent (Fig 1c).

**Fig 2:**
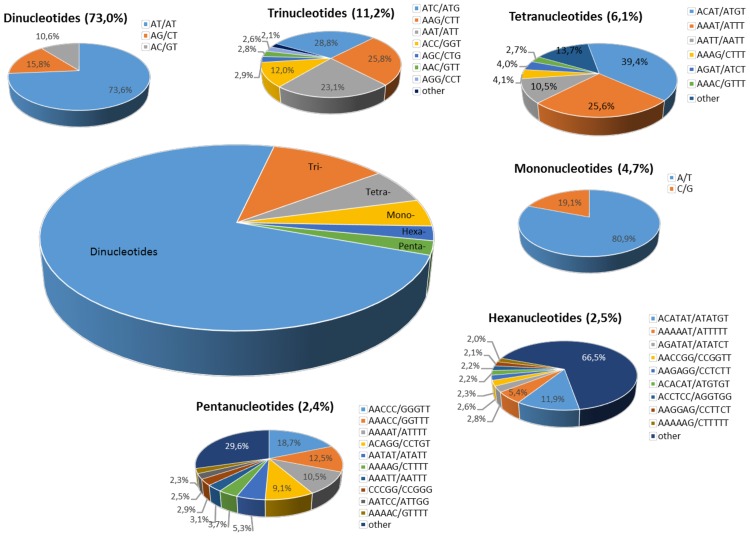
The distribution of the major repeat types in the globe artichoke genome.

After grouping the motifs, as suggested by Jurka and Pethiyagoda [6] (for example, (TTC)_n_, (TCT)_n_, (CTT)_n_, (AGA)_n_ and (GAA)_n_ were considered as equivalent when read in different reading frames and on the complementary strand), a total of 428 SSR motif types were represented. These included all of the possible base combinations of mono- (the number is 2), di- (4) tri- (10) and tetranucleotide (33), while there were 95 and 284 types of penta- and hexanucleotide repeats, respectively (Table 1). We also conducted a detailed analysis of individual repeat motifs for each type of SSR found in the globe artichoke genome sequence (Fig 2, S3 Table). Among the mononucleotide types A/T predominated heavily (80.9%), as did AT/TA among the dinucleotide types (73.6%); the most frequently occurring motif types among the trinucleotide SSRs was ATC/ATG (28.8%), among the tetranucleotide types ACAT/ATGT (39.4%), among the pentanucleotide types AACCC/GGGTT (18.7%) and among the hexanucleotide types ACATAT/ATATGT (11.9%). The longest SSR detected harbored 84 ACAT repeats, and the highest number of repeats present at a locus was a string of 166 AT's. AT/TA was not just the predominant dinucleotide motif, but was also the most frequent motif globally, represented in 53.7% of the SSRs; in contrast, CG/CG was very rare (0.002%). Among the trinucleotides, ATC, AAG and AAT were the most abundant motifs (present in ~77% of SSRs), whereas the GC-rich motifs ACG and CCG were the least common. Similarly, the AT rich motifs ACAT, AAAT and AATT dominated the set of tetranucleotide SSRs (present in ~75% of SSRs), and the motifs AACCC, AAACC, AAAAT and ACAGG together represented 53.7% of the pentanucleotide SSRs. The only two hexanucleotide motifs featuring a frequency >5% were ACATAT and AAAAAT (Fig 2).

### The intra-chromosomal distribution of SSRs

**Fig 3:**
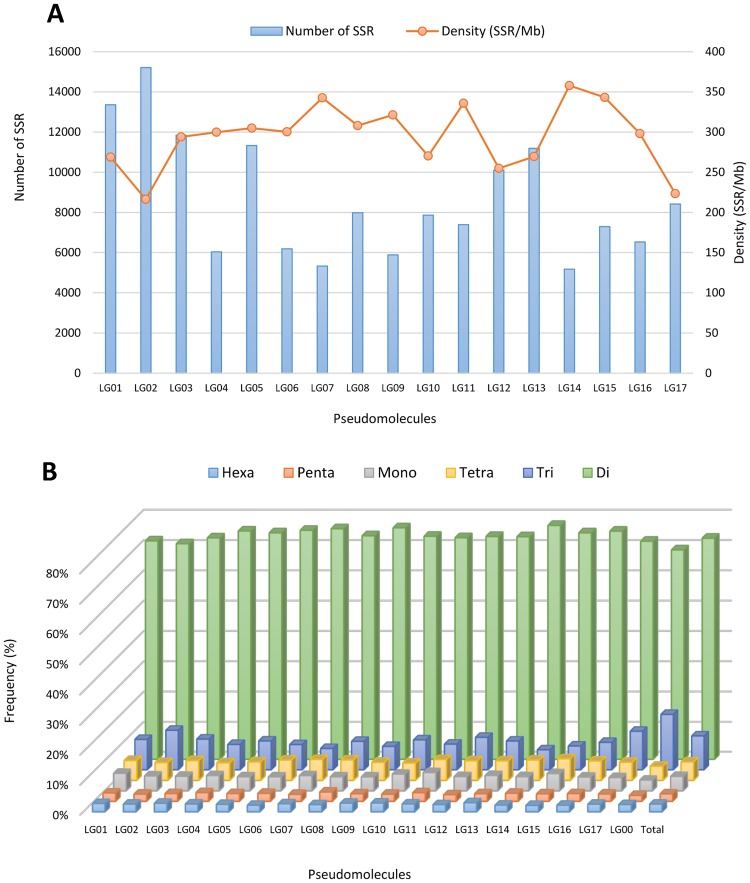
The intra-chromosomal distribution of SSRs. (A) The comparison of perfect SSR number/density. (B) The frequency of mono- to hexanucleotide motifs in the 17 linkage groups of globe artichoke.

The SSR loci identified were further classified on the basis of their repeat motif and distribution over each pseudomolecule (Table 2, S4 Table). Although the genome sequence has been organized into 17 pseudomolecules, representing the haploid globe artichoke set, ~17% of the SSRs were detected in not anchored scaffolds (LG00). Of the remaining ~83%, the mean number per LG of perfect and imperfect SSRs was, respectively, 8,657 and 10,926. Although the mean density of perfect SSRs across the entire genome was calculated as 244.5 SSRs per Mbp, this value increased to 280.0 SSRs per Mbp when only the anchored scaffolds were considered. The greatest number of SSRs (15,212 perfect, 19,282 imperfect) was assigned to the longest LG (LG02, 70.34 Mbp), and the lowest (5,181 perfect, 6,518 imperfect) to the shortest (LG14, 14.48 Mbp), resulting in a range of perfect SSR density from 216.3 to 357.9 per Mbp (Table 2). Although in general the shorter the LG, the greater the SSR density, LG03, LG05 and LG17 were exceptions (Fig 3a). The intra-chromosomal distribution of motif types reflected that present in the genome as a whole (Fig 3b) dinucleotide repeats were the commonest and the penta-/hexanucleotides the rarest. The di- and trinucleotide exhibited the highest level of variation among LGs, with LG02 showing the lowest percentage for di- (71.2%) and highest for trinucleotides (13.1%), while in LG14 an opposite trend was found (di- 77.2%; tri- 6.6%). When considered on a chromosome-by-chromosome basis, A was the most abundant mononucleotide and AT the most common dinucleotide repeat (as in the genome as a whole). Meanwhile among the trinucleotide repeats, ATC, AAG, AAT or ACC were the most abundant, among the tetranucleotide repeats the three most frequent on each LG were ACAT, AAAT and AATT (in that order), and the four repeats AACCC, AAACC, AAAAT and ACAGG together represented about half of the pentanucleotide SSRs present, albeit their relative contribution varied from LG to LG. Finally, ACATAT was the most frequent hexanucleotide motif in each LG, contributing between 7.5% and 17.2% (S3 Table).

**Table 2:** The chromosome-by-chromosome distribution of perfect, compound and imperfect SSRs.

Linkage groups	Total Mbp	Perfect								Compound		Imperfect	
		Mono-	Di-	Tri-	Tetra-	Penta-	Hexa-	Total	SSRs/Mbp	Total	SSR/Mbp	Total	SSRs/Mbp
**LG01**	49.71	772	9,637	1,345	872	366	368	**13,360**	268.8	**2,714**	54.6	**17,309**	348.2
**LG02**	70.34	747	10,834	1,993	893	359	386	**15,212**	216.3	**3,150**	44.8	**19,282**	274.1
**LG03**	40.26	566	8,660	1,206	774	302	335	**11,843**	294.2	**2,581**	64.1	**14,919**	370.6
**LG04**	20.15	304	4,553	507	349	171	155	**6,039**	299.7	**1,304**	64.7	**7,593**	376.9
**LG05**	37.16	523	8,476	1,081	688	288	275	**11,331**	304.9	**2,552**	68.7	**14,238**	383.1
**LG06**	20.61	284	4,683	517	414	161	133	**6,192**	300.4	**1,472**	71.4	**7,627**	370.0
**LG07**	15.56	267	4,059	376	367	122	140	**5,331**	342.6	**1,209**	77.7	**6,693**	430.1
**LG08**	25.92	369	5,894	752	537	244	189	**7,985**	308.0	**1,808**	69.7	**9,888**	381.4
**LG09**	18.33	270	4,502	456	349	145	167	**5,889**	321.3	**1,277**	69.7	**7,380**	402.7
**LG10**	29.10	434	5,797	781	447	184	227	**7,870**	270.4	**1,700**	58.4	**9,974**	342.7
**LG11**	22.01	447	5,410	629	508	208	191	**7,393**	336.0	**1,522**	69.2	**9,298**	422.5
**LG12**	39.65	474	7,436	1,087	651	217	238	**10,103**	254.8	**2,199**	55.5	**12,575**	317.2
**LG13**	41.51	568	8,227	1,066	718	279	334	**11,192**	269.6	**2,353**	56.7	**14,165**	341.3
**LG14**	14.48	241	4,002	342	347	136	113	**5,181**	357.9	**1,219**	84.2	**6,518**	450.3
**LG15**	21.26	414	5,456	574	519	178	153	**7,294**	343.0	**1,572**	73.9	**9,256**	435.3
**LG16**	21.91	295	4,921	594	416	165	141	**6,532**	298.1	**1,485**	67.8	**8,216**	374.9
**LG17**	37.69	366	6,066	1,077	502	201	211	**8,423**	223.5	**1,798**	47.7	**10,808**	286.8
**LG00**	199.02	1,052	20,777	5,526	1,391	576	715	**30,037**	150.9	**5,833**	29.3	**38,323**	192.6
**Total**	**724.67**	**8,393**	**129390**	**19,909**	**10,742**	**4,302**	**4,471**	**177,207**	**244.5**	**37,748**	**52.1**	**224,062**	**309.2**

### Genic SSRs

**Fig 4:**
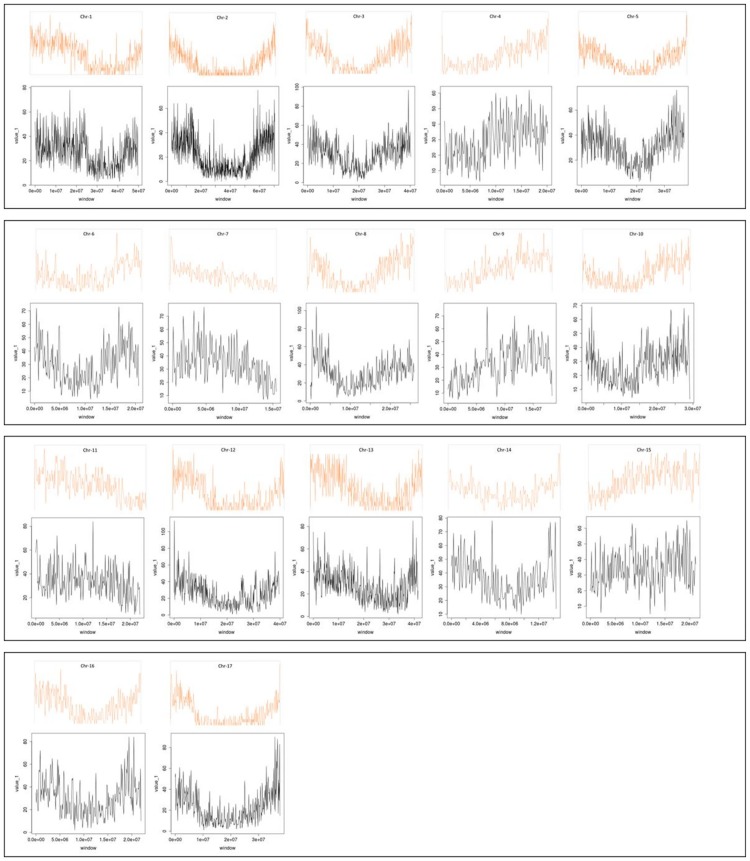
The distribution of SSRs along each of the 17 globe artichoke linkage groups. The orange trend line tracks gene density, and the boxed black trend line the density of perfect SSRs.

**Fig 5:**
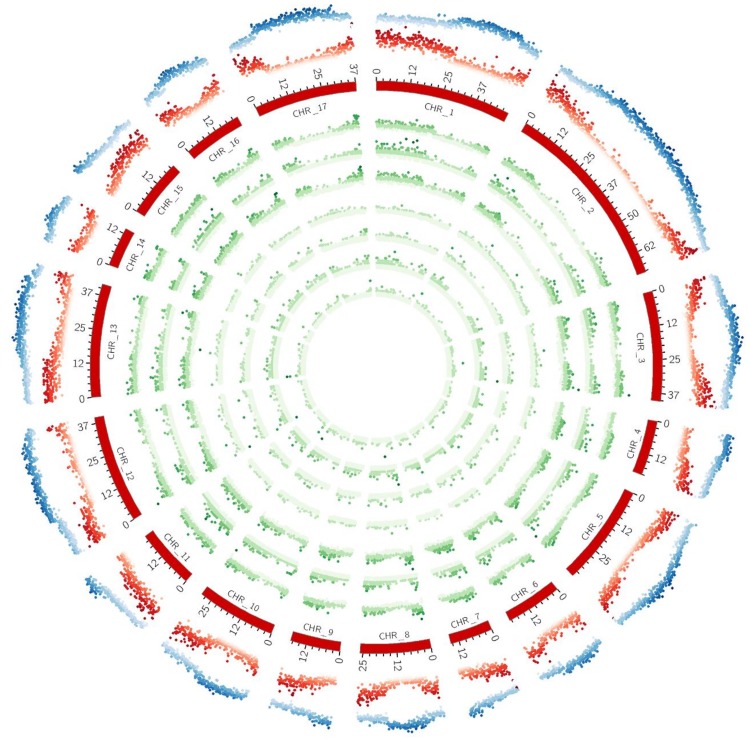
Circos diagram depicting the chromosome-scale SSR distribution (perfect). In green, from outside to inside: all SSRs, SSRs formed by mono-, di-, tri-, tetra-, penta- and hexanucleotides. In red: gene density. In blue: repeat density.

Based on the available and assembled pseudomolecules of globe artichoke, the genomic distribution of SSRs and the relationship with annotated genomic components (gene space) was analyzed. The SSRs embedded in genic sequence were concentrated towards the chromosome ends (Fig 4), irrespective of motif type and in accordance with the gene space distribution (Fig 5). In all, 4,761 (2.7% of the total) perfect and 6,583 (2.9%) imperfect SSRs were identified as present in genic sequence, distributed within 3,781 genes (about 14% of the full gene complement) (Table 3). As the globe artichoke gene space is estimated to span 146.5 Mbp [4], the overall gene space SSR density was estimated to be 32.5 and 44.9 SSRs per Mbp for perfect and imperfect motifs respectively. The most common motifs represented (among the perfect SSRs: 54.9%, among the imperfect SSRs: 42.4%) were the trinucleotides, they were followed by dinucleotide (21.4%) within the perfect motif, while the hexanucleotides represented the second most frequent type within the imperfect ones (22.5%) (Table 3). Repeat numbers were lower than in the genome as a whole, with 84.0% of SSRs composed of ten or less repeats and less than 2% harboring 20 or more repeats. A total of 275 motif types were represented, the most common being ACC/GGT (14.2%), followed by AG/CT (12.0%), AAG/CTT (11.8%), ATC/ATG (11.1%) and AT/AT (9.6%); none of the other motifs was associated with a frequency above 5%. The intra-chromosomal distribution of the genic SSRs is summarized in S5 Table. As for the global set of SSRs, the highest number of genic SSRs was found on the longest LG, and the lowest on the shortest LG.

**Table 3:** Variation in repeat length among genic globe artichoke perfect and imperfect SSRs.

SSR Type	Perfect motif			Imperfect motif		
	Count	%	Density (SSRs/Mbp)	Count	%	Density (SSRs/Mbp)
Mono-	108	2.3%	0.74	112	1.7%	0.76
Di-	1,020	21.4%	6.96	1,243	18.9%	8.48
Tri-	2,614	54.9%	17.84	2,794	42.4%	19.07
Tetra-	327	6.9%	2.23	462	7.0%	3.15
Penta-	151	3.2%	1.03	493	7.5%	3.37
Hexa-	541	11.4%	3.69	1,479	22.5%	10.10
**Total/mean**	**4,761**	**100.0%**	**32.50**	**6,583**	**100.0%**	**44.94**

**Fig 6:**
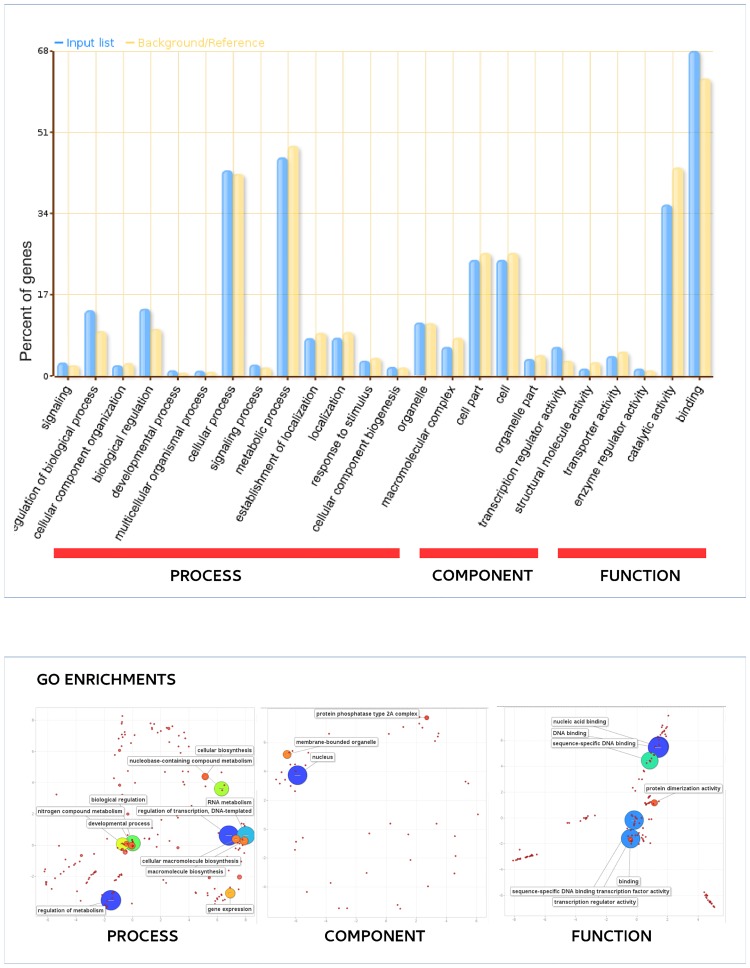
Functional analysis (gene ontology) of the set of globe artichoke genes containing SSRs. (A) GO categorization. The blue bars indicate input genes, while the green bars indicate background genes. (B) REVIGO summary of “biological process”, “molecular function” and “cellular component” enriched terms. The bubble size is proportional to the log_10_ (p value) of enrichment analyses and its color is also a function of log_10_(p value) of enrichment analyses (blue: low, -red: high p value). The *x* and *y* axes reflect semantic similarity according to the REVIGO algorithm (similar GO terms appear close together).

The genic repeated motifs were located in 3,781 genes (14.11% of the total genes). The genes harboring one or more SSRs were grouped into the three GO main categories (“biological processes” (BP), “molecular functions” (MF) and “cellular components” (CC)), and thereafter in the 24 sub-GO categories. Over-representation was noted for a number of genes (Fig 6, S6 Table). Those allocated to BP belonged to the sub-categories “gene expression” (GO:0010467), “regulation of gene expression” (GO:0010468), “regulation of transcription, DNA-dependent” (GO:0006355), “cellular macromolecule metabolic process” (GO:0044260). Similarly on the basis of MF, enrichment was observed for “nucleic acid binding” (GO:0003676), “sequence-specific binding level (GO:0043565), “transcription regulator activity” (GO:0030528) and “protein dimerization activity” (GO:0046983). Finally with respect to CC, the only identified sub-category was “nucleus” (GO:0005634).

### *CyMSatDB* construction, content and system architecture

**Fig 7:**
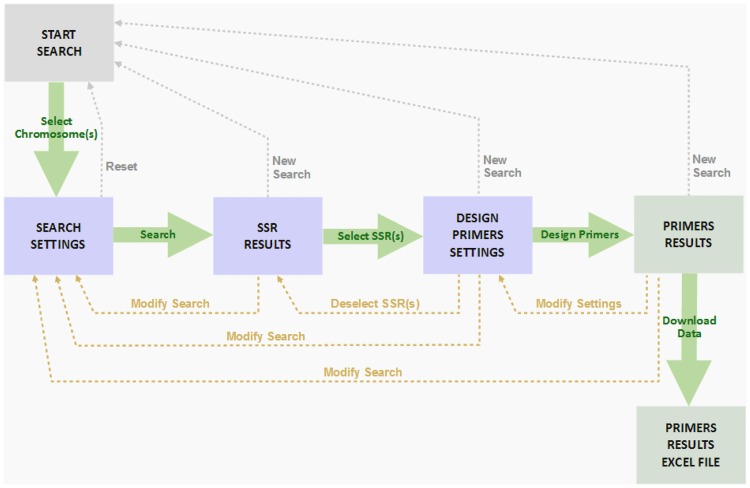
Work flow for the *CyMSatDB* user.

**Fig 8:**
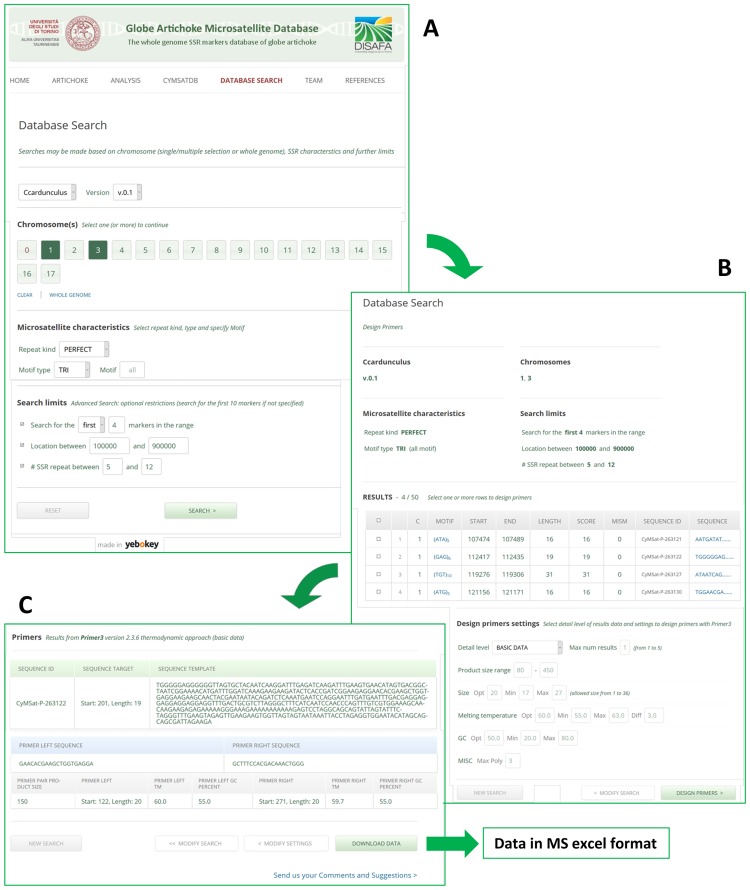
A worked example of an SSR search and primer design using *CyMSatDB*. (A) Settings given for chromosome selection and SSR search. (B) The SSR output and settings given for the design of primers. (C) Suggested primers and the downloading of the result.

The public domain *CyMSatDB*, available at www.artichokegenome.unito.it/cymsatdb/, provides a searchable interface to the current set of SSR data. Fig 7 provides a flow of user operation, interaction and various features and utilities. The database provides browsable access to all the SSRs identified in the globe artichoke genome; SSRs can be retrieved on the basis of simple characteristics, such as “genomic location” (chromosome number), “SSR feature” (whole genomic *or* only genic SSR), “repeat kind” (perfect *vs* imperfect), or advanced characteristics, such as “motif type” (mono- to hexa-nucleotide), “specific motif sequence”, “repeat number”, “specific genomic location”. Multiple parameters can be also combined to search for a specific set of SSRs as per user requirement. Microsatellites can be mined based on the choice of chromosome, where more than one chromosome may also be selected (Fig 8a). The flexibilities provided will enable researcher to select markers of choice at desired genomic interval, by limiting the search based on chromosomal location as well as the number of markers in that range.

The output lists all the SSRs meeting the user-selected parameters(s) in tabular form, along with SSR identifiers, LG number, motif type and length, genomic location (start and end position), SSR length and an option for downloading the flanking sequences (Fig 8b). Primers can be readily designed to amplify a selected SSR primers, using the Primer3 standalone tool. The user may go for primer designing as a default setting or modify the standard Primer3 parameters. The ‘Design Primers’ button directs the use to a list of up to five possible primer pairs, with their melting temperatures (Tm), their GC content and the expected length of the amplicon. The primer information can be readily exported into Microsoft Excel (Fig 8c).

### Marker validation

Validating SSR markers generally requires using them to prime PCRs containing a set of relevant template DNA. Here, an *in silico* validation was performed, taking advantage of extant sequence data acquired from two contrasting globe artichoke accessions. The 38,726 unigene set derived from 'Romanesco C3' [7] enabled the validation of about a half (1,644/3,265) of the markers, while the 19,055 unigene set prepared from assembled ESTs of 'Green Globe' was used to validate a slightly higher proportion of the assays (837/1,426). All the validated SSRs belonged to genic loci.

## Discussion

### Globe artichoke microsatellite data projects

The first set of globe artichoke SSRs to have been developed was based on a combination of scanning extant sequence and analyzing a genomic library deliberately enriched for SSR-containing sequence [8]. The set of SSRs discovered in this way were later supplemented from the output of a “microsatellite amplified library” approach [9], the technique represents a combination of AFLP and a primer extension-based enrichment and provided a rapid means to increase the efficiency of microsatellite identification by avoiding the need of a hybridization enrichment step. *C*. *cardunculus* microsatellites were even further obtained by applying a microsatellite-AFLP procedure [10], and from genomic libraries enriched for (GT)_n_, (GA)_n_, (TCT)_n_, (TGT)_n_, (GAG)_n_, (GTG)_n_, (TGA)_n_, (AGT)_n_, (GCT)_n_ and (GCC)_n_ [11]. The most recent and extensive set of globe artichoke microsatellite was developed by Scaglione *et al*. [12]; over 4,000 potential SSR loci were identified from expressed sequence tags (EST) and sample of them showed to be polymorphic among the parents of two mapping populations. The utility of the many SSR assays derived in recent years has been proven in the context of linkage mapping, diversity analysis and phylogenetic analysis [1, 11, 13, 14]. Marker-rich linkage maps made possible using these resources [15] have enabled a number of key agronomic traits to be subjected to genetic analysis [16–17]. The availability of a very large set of microsatellite markers, distributed throughout the genome, such as those described in this publication facilitates the development of high-resolution maps and/or rapid saturation of specific map regions, which are instrumental for positional gene cloning and comparative mapping studies.

### The SSR content of the globe artichoke genome and cross-species comparison

The major goal of the present exercise was to identify a large set of SSR loci, which by placing restrictions on both the repeat length (>15 nt) and the repeat number (at least four), had a increased chance of being informative, since such loci are more *prone to* strand slippage [18]. The resulting set of loci were spread throughout the genome, with a larger number of loci present on the longer chromosomes, as would be expected if their distribution was truly random. Among the perfect SSRs, there was an inverse relationship between their frequency and the number of repeat units (i.e, loci harboring many units were less abundant than those harboring few), a relationship which has also been noted in a number of other genomes [19–21].

**Fig 9:**
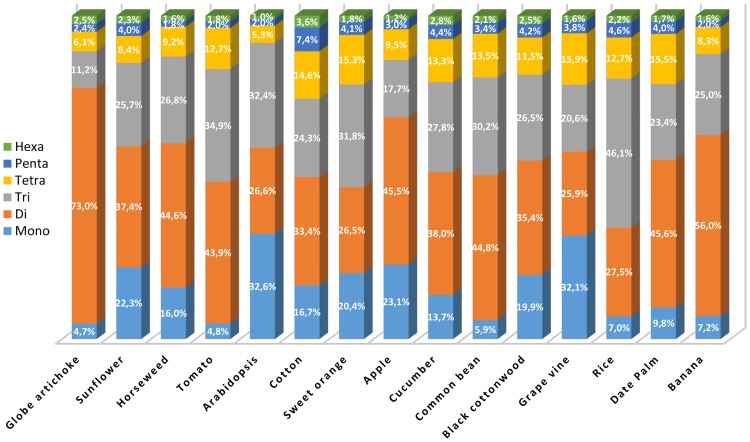
The representation of different SSR motifs across 15 plant genomes.

The content and distribution of SSRs in the globe artichoke genome was compared with that present in 14 other plant genomes (S1 Table). In each of these species, the global content of perfect SSRs composed of mono- to hexanucleotides, with ≥4 repeats and with a length of at least 15 nt was identified. The globe artichoke genome included almost twice as many perfect SSRs as in tomato (88,781), four times as many as in sweet orange (43,147) and ten times as many as in *Arabidopsis* (17,289) (Table 4), but fewer than in sunflower (269,635), a fellow Compositae species. The total length of sequence represented by repeats in globe artichoke (~4.4 Mbp) was equivalent to 0.61% of the assembled genome, a proportion comparable to that in both grapevine (0.67%) and black cottonwood (0.57%), but was significantly higher than in the other 12 species (Table 4). With respect to compound SSRs, the number present in globe artichoke was ~21% that of the perfect SSRs, a similar proportion to that in tomato (27.6%), overcome only by the number detected in sunflower (36.7%). A negative relationship between genome size and SSR density has been reported by Morgante *et al*. [22]. Even though the largest genomes analysed here (cotton and sunflower) indeed displayed the lowest SSR density, the SSR density in globe artichoke (244.5 per Mbp) was more than double that in tomato (113.6 per Mbp), in spite of their comparable genome sizes, but analogous to the one of grape wine and black cottonwood (263.7 and 227.1 SSRs/Mbp, respectively), which own smaller genomes. *Arabidopsis* also deviated from the trend, as previously noted by Cavagnaro *et al*. [20] its genome size is small, but its SSR density is lower (144.4 per Mbp) than in both horseweed (double its genome size) and globe artichoke (six times its size). In respect to the other genomes in study, another signature of the globe artichoke genome was the high frequency (73%) of dinucleotide perfect motifs. Together, mono-, di- and trinucleotide repeats formed the bulk of the SSR content in all 15 genomes analysed (ranging from 74% in cotton to 92% in *Arabidopsis*). In globe artichoke, tomato, common bean, date palm and banana, dinucleotide motifs predominated, followed by the tri-, tetra- and mononucleotide ones (Fig 9). However, in both of the other two Compositae species (sunflower and horseweed), the frequency of mononucleotide repeats was higher than that of tetranucleotide repeats. The distribution of SSR types in globe artichoke was most unlike that in *Arabidopsis* and grapevine (where mononucleotide motifs predominated), and in sweet orange and rice (where trinucleotide motifs were by far the commonest type).

**Table 4:** A comparative survey of perfect SSRs across 15 plant species.

Genome	Analyzed sequences (Mbp)	Perfect SSRs					Compound SSRs	
		Kinds	Count	Density (SSRs/Mbp)	Cumulative (Mb)	Cumulative (%)	Count	%
**Globe artichoke**	724.7	428	177,207	244.5	4.41	0.61%	37,748	21.3%
**Sunflower**	3602.3	405	269,635	74.9	9.05	0.25%	99,039	36.7%
**Horseweed**	326.2	268	62,185	190.7	1.48	0.45%	4,807	7.7%
**Tomato**	781.7	278	88,781	113.6	2.58	0.33%	24,516	27.6%
**Arabidopsis**	119.7	172	17,289	144.4	0.37	0.31%	763	4.4%
**Cotton**	1694.6	378	115,761	68.3	2.84	0.17%	7,686	6.6%
**Sweet orange**	319.2	317	43,147	135.2	0.93	0.29%	3,644	8.4%
**Apple**	881.3	346	146,282	166.0	3.68	0.42%	15,924	10.9%
**Cucumber**	203.1	318	40,391	198.9	0.94	0.47%	3,034	7.5%
**Common bean**	521.1	291	55,289	106.1	1.33	0.26%	4,884	8.8%
**Black cottonwood**	434.1	361	98,604	227.1	2.42	0.56%	8,994	9.1%
**Grape vine**	486.2	312	128,216	263.7	3.25	0.67%	15,921	12.4%
**Rice**	374.5	358	64,927	173.4	1.68	0.45%	8,170	12.6%
**Date palm**	556.5	311	79,452	142.8	2.00	0.36%	12,636	15.9%
**Banana**	472.2	336	78,444	166.1	2.34	0.50%	13,018	16.6%

Grouping repeats in the manner suggested by Jurka and Pethiyagoda [6] revealed 493 motifs across the set of 15 genomes, of which fully 428 were represented in globe artichoke; the next most motif-diverse genome was sunflower (405 motifs) (Table 4). All the possible mono- (2) di- (4) and trinucleotide (10) were present in all 15 genomes, but there were interspecific differences with respect to the motif types present in the higher level repeat units. As in most plant species, the base composition of the globe artichoke SSR motifs was AT rich (S7 Table). With respect to the mononucleotide motifs, A/T predominated in 14 of the 15 species (the exception was rice, where the proportion was only ~38%). Among the dinucleotide motif types, AT/TA was the most frequent, except in date palm, where the commonest motif was AG/CT. The frequency of the CG/GC motif rose above 1% only in rice. It has been suggested by Cavagnaro *et al*. [20] that AT rich repeats predominate in dicotyledonous but not in monocotyledonous species, and that this difference reflects their genomes' nucleotide composition: the mean GC% content of dicotyledonous species is 34.6%, while that of monocotyledonous species is 43.7%. However, this difference could not satisfactorily account for the disparate frequency of certain motifs between these two phylogenetic classes. Rather uniquely, the commonest tri- and pentanucleotide motifs in globe artichoke were ATC and AACCC, (and not AAT and AAAAT) while an abundance of the tetra- ACAT repeats was found only in globe artichoke and sunflower. ACATAT, the only hexanucleotide repeat occurring at a frequency >10%, unexpectedly happened also to be the most frequent one in the dicotyledonous species sampled, rice and date palm. In common with Cheng *et al*. [19], certain motifs were identified as being species-specific: these were associated with six of the 15 species all of them are hexanucleotide with very low frequencies (S8 Table). The hexanucleotides ACGTAG/ACGTCT and AGGGCC/CCCTGG were unique to globe artichoke, while AGCGAT/ATCGCT was restricted to the three Compositae species globe artichoke, sunflower and horseweed.

### Genic SSRs in globe artichoke

The overall microsatellite density in globe artichoke genes was significantly lower than in genomic sequence and their distribution was coherent with that of genes and opposite to that of the repetitive elements in all the pseudomolecules analyzed (Figs 4 and 5), in agreement with the reports of Morgante *et al*. [22]. As has been shown for other genomes [22–24], genic SSRs tend to be composed of either tri- or hexanucleotides. A likely reason for this prevalence is that, in coding regions, frameshift mutations generated by replication slippage are likely to experience negative selection: alterations in the repeat number of a tri- or hexanucleotide repeat, depending on the SSR's position within the sequence, do not induce frameshift. With respect to the surviving dinucleotide motifs in globe artichoke, while AT/TA repeats tend to prevail in non-transcribed regions, AG/CT is commonest in genic sequence, which is the case in other species as well [20, 22]. There was generally a preference for GC rich motif in the trinucleotide repeats present in genic DNA: the GC content was ~48%, whereas it was only ~32% in the global set of trinucleotide repeats. SSRs embedded in genic DNA are generally more likely to be conserved across (related) species boundaries than those in non-genic DNA, a feature which makes them attractive for comparative mapping [25]. On the other hand, since genic DNA is under positive selection, these SSRs are typically less polymorphic. Given that a high repeat number is correlated with allelic variation, it was therefore unsurprising to find that nearly 5% of the global globe artichoke SSRs harbored >20 repeats, while this proportion among the genic SSRs was under 2%.

An analysis of the 3,781 SSR-containing genic segments in the globe artichoke genome revealed that certain gene regulation functions (GO:0010467, GO:0010468, GO:0006355) featured frequently (Fig 6, S6 Table). A prominent class of genes were those encoding transcription factors (GO:0003676, GO:0043565, GO:0030528, GO:0005634). A similar concentration of SSRs within specific classes of genic sequence has been observed in other species [26–30]. The implications of transcription factor sequences harboring an SSR have been alluded to elsewhere [30] and should be addressed in future analyses of diversification within globe artichoke.

### *CyMSatDB* features and utility

The *Cynara cardunculus* MicroSatellite DataBase (*CyMSatDB*) is a user-friendly, freely accessible tool that offers chromosome wise, as well as location wise, search of primers. In particular, the advanced options for search and primer design eliminates the need of manual primer designing using a separate tool/server, and are displayed on an effective and responsive interface developed in PHP. *CyMSatDB* was specifically designed to work efficiently with any mobile device and smartphone, so to rapidly access from iphone, android, ipad, and tablet, easily searched and for custom primer designing. When displayed on a smaller device, *CyMSatDB* will automatically resize and optimize to look optimal also for small devices. Designed for domain experts, this interface implements Error prevention and Auto-correction functions. In particular it: (i) reminds the default values / ranges for each field; (ii) auto-corrects data on key-press instead of on mouse-out; (iii) explains errors using detailed descriptive tooltips; (iv) signals success when the field is correctly completed. The system offers two kinds of data export of the designed primers: a synthetic version showing only the most relevant data and a complete one. The data download provides an excel file specifically formatted for a higher readability. A management interface is also available for administrators to ease the data import in the system. The conversion of the SciRoKo output into CyMSatDB compatible data is made simple through immediate interface operations instead of using command line scripts.

In addition to understanding the characteristics of SSR distribution, the development of molecular markers for globe artichoke was an important driver of this study. In an *in silico* validation exercise, achieved by exploiting the availability of the assembled transcriptome of two globe artichoke accessions, over 50% of primer pairs were predicted to be functional. This was considered a positive result, since in a similar study Iquebal *et al*. [31] reported that only 11% of primer pairs could be validated in this way. One possible reason underlying a failed validation may reflect the presence of a large intron(s) between the pair of primer sites, because the sequence used for primer design was exonic; a second possibility arises from the reference sequence having been acquired from a highly inbred accession, since the frequency of null SSR alleles in the globe artichoke genome is known to be rather high [10]. The different genetic structure of the varieties used to assemble unigenes [7, 12] might have affected the results as well. Nevertheless, the first globe artichoke microsatellite database may provide key information also for the completion of the genome anchoring of unmapped scaffolds.

Genic SSRs retain some relevance as functional markers in their own right, as their inclusion in transcripts has been documented to have an effect on gene expression/function in both human [32, 33] and *Arabidopsis* [34]. Of particular note is the presence of SSRs within loci encoding an miRNA [35–37], the products of which are of great significance to gene expression. Several putative miRNA-SSRs have been identified in the globe artichoke genome, most of which harbor AT/TA dinucleotides. The presence of SSRs within such loci represents an application for SSRs, beyond their conventional use as simple genetic markers [4]. The database provides the resources required to access relevant DNA sequences to address the functionality (if any) of genic SSRs. The marker information housed in *CyMSatDB* will be of high value for linkage mapping and for facilitating the marker saturation of genomic regions. Several plug-ins have been implemented to generate primers at a user-defined chromosomal location. The database could be used to design assays sampling more than one SSR locus in a single PCR, which require both compatibility with respect to primer annealing temperature and a non-overlap in amplicon size. Such multiplex assays are of particular relevance to varietal identification, especially in the context of plant variety protection and varietal release.

## Conclusions

The global analysis of the globe artichoke genome has revealed some 177,000 perfect and 224,000 imperfect SSR loci. The same search parameters were also used to extract the SSR content of 14 plant species. Due to the algorithms used for assembly of reads into contigs and scaffolds, the globe artichoke genome assembly used in our study most likely corresponds to the single/low copy fraction of the genome, while left-aside unassembled sequence reads represent most of the repetitive fraction, perhaps explaining the rather high frequency of the SSRs revealed (Table 4). Indeed, it has been previously reported that the distribution of SSRs in plant genomes is not random and predominates in non-repetitive sequences [22, 38]; thus our data, mostly generated from non-redundant genomic sequences, might be an overestimate of the SSR density in the globe artichoke genome. The *CyMSatDB* database houses a full set of information regarding both genic and non-genic perfect and imperfect SSR loci. Its intuitive web interface and its provision of a customized primer design tool offers biologists and breeders interested in globe artichoke a highly flexible tool. The database facilitates the selection of SSRs of a particular type or from a specific region in the genome. It will be continuously updated as resequencing data of globe artichoke and/or cultivated cardoon genotypes will become available, allowing the *in silico* identification polymorphic SSRs as a result repeat-length expansion/contraction. Moreover, since some part of the SSRs could be transferable to related taxa within the Asteraceae family, the database might be exploitable by a wider community of researchers.

## Supporting Information

S1 Table List of the 16 genomes analysed for SSR identification in the present study. (XLSX)

S2 Table Frequency of the main identified SSR motifs (considering sequence complementary) classified by number of repeats. (XLSX)

S3 Table Frequency and distribution of main repeat motif types (considering sequence complementary) in 17 pseudomolecules and unmapped scaffolds of the globe artichoke genome. (XLSX)

S4 Table The chromosome-by-chromosome distribution of imperfect microsatellite in the globe artichoke genome. (XLSX)

S5 Table The chromosome-by-chromosome distribution of perfect and imperfect microsatellite in the globe artichoke gene. (XLSX)

S6 Table GO categorisation of the globe artichoke genes containing SSRs and over-representation of GO-terms. The significance level threshold was placed at p-value < e−5 and false.
(XLSX)

S7 Table Frequency of all mono-/di-/trinucleotide SSR motif and of the ten most frequent tetra-/penta-/hexanucleotide SSR motif in all investigated genomes. (XLSX)

S8 Table Species-specific hexanucleotide SSR motifs and relative frequencies within hexa-SSR in each genome. (XLSX)

## References

[1] Portis E, Mauromicale G, Barchi L, Mauro R, Lanteri S. Population structure and genetic variation in autochthonous globe artichoke germplasm from Sicily Island. Plant Science 2005a;168:1591–1598.

[2] Mauro R, Portis E, Acquadro A, Lombardo S, Mauromicale G, Lanteri S. Genetic diversity of globe artichoke landraces from Sicilian small-holdings: implications for evolution and domestication of the species. Cons Genet. 2009;10:431–440.

[3] FAOSTAT 2013. Available: http://faostat.fao.org/

[4] Scaglione D, Reyes-Chin-Wo S, Acquadro A, Froenicke L, Portis E, Beitel C, et al The genome sequence of the outbreeding globe artichoke constructed de novo incorporating a phase-aware low-pass sequencing strategy of F1 progeny. Scientific Reports. 2016;6:19427 10.1038/srep19427 26786968 PMC4726258

[5] Xu J, Liu L, Xu Y, Chen C, Rong T, Ali F, et al Development and characterization of simple sequence repeat markers providing genome-wide coverage and high resolution in maize. DNA research. 2013; 20:497–509. 10.1093/dnares/dst026 . 23804557 PMC3789560

[6] Jurka J, Pethiyagoda C. Simple repetitive DNA sequences from primates: compilation and analysis. J Mol Evol. 1995;40:120–126. 7699718 10.1007/BF00167107

[7] Scaglione D, Lanteri S, Acquadro A, Lai Z, Knapp SJ, Rieseberg L, Portis E. Large-scale transcriptome characterization and mass discovery of SNPs in globe artichoke and its related taxa. Plant Biotech J. 2012;10:956–969. 10.1111/j.1467-7652.2012.00725.x 22849342

[8] Acquadro A, Portis E, Lanteri S. Isolation of microsatellite loci in artichoke (*Cynara cardunculus* L. var. *scolymus* L.). Mol Ecol Notes. 2003;3:37–39.

[9] Acquadro A, Portis E, Albertini E, Lanteri S. M-AFLP based protocol for microsatellite loci isolation in *Cynara cardunculus* L. (Asteraceae). Mol Ecol Notes. 2005a;5:272–274.

[10] Acquadro A, Portis E, Lee D, Donini P, Lanteri S. Development and characterisation of microsatellite markers in *Cynara cardunculus* L. Genome. 2005b;48:217–225. 15838543 10.1139/g04-111

[11] Acquadro A, Lanteri S, Scaglione D, Arens P, Vosman B, Portis E. Genetic mapping and annotation of genomic microsatellites isolated from globe artichoke. Theor Appl Genet. 2009;118:1573–1587. 10.1007/s00122-009-1005-6 19326092

[12] Scaglione D, Acquadro A, Portis E, Taylor CA, Lanteri S, Knapp SJ. Ontology and diversity of transcript-associated microsatellites mined from a globe artichoke EST database. BMC Genomics. 2009;10:454 10.1186/1471-2164-10-454 19785740 PMC2760586

[13] Lanteri S, Acquadro A, Comino C, Mauro R, Mauromicale G, Portis E. A first linkage map of globe artichoke (*Cynara cardunculus* var. *scolymus* L.) based on AFLP, S-SAP, M-AFLP and microsatellite markers. Theor Appl Genet. 2006;112(8):1532–1542. 16565844 10.1007/s00122-006-0256-8

[14] Portis E, Barchi L, Acquadro A, Macua JI, Lanteri S. Genetic diversity assessment in cultivated cardoon by AFLP (amplified fragment length polymorphism) and microsatellite markers. Plant Breed. 2005b;124:299–304.

[15] Portis E, Scaglione D, Acquadro A, Mauromicale G, Mauro R, Knapp S, Lanteri S. Genetic mapping and identification of QTL for earliness in the globe artichoke/cultivated cardoon complex. BMC Research Notes. 2012;5:252 10.1186/1756-0500-5-252 22621324 PMC3434057

[16] Portis E, Mauro RP, Barchi L, Acquadro A, Mauromicale G, Lanteri S. Mapping yield-associated trait QTL in globe artichoke. Mol Breed. 2014;34:615–630.

[17] Portis E, Mauro RP, Acquadro A, Moglia A, Mauromicale G, Lanteri S. The inheritance of bract pigmentation and fleshy thorns on the globe artichoke capitulum. Euphytica. 2015;206:523–531.

[18] Whittaker JC, Harbord RM, Boxall N, Mackay I, Dawson G, Sibly RM. Likelihood-based estimation of microsatellite mutation rates. Genetics. 2003;164(2):781–787. 12807796 10.1093/genetics/164.2.781 PMC1462577

[19] Cheng J, Zhao Z, Li B, Qin C, Wu Z, Trejo-Saavedra DL et al A comprehensive characterization of simple sequence repeats in pepper genomes provides valuable resources for marker development in *Capsicum*. Sci Rep. 2016;6:18919 10.1038/srep18919 26739748 PMC4703971

[20] Cavagnaro PF, Senalik DA, Yang L, Simon PW, Harkins TT, Kodira CD et al Genome-wide characterization of simple sequence repeats in cucumber (*Cucumis sativus* L.). BMC Genomics. 2010;11:569 10.1186/1471-2164-11-569 20950470 PMC3091718

[21] Shi J, Huang S, Fu D, Yu J, Wang X, Hua W, et al Evolutionary Dynamics of Microsatellite Distribution in Plants: Insight from the Comparison of Sequenced Brassica, Arabidopsis and Other Angiosperm Species. Vinatzer BA, ed. PLoS ONE. 2013;8(3):e59988 10.1371/journal.pone.0059988 23555856 PMC3610691

[22] Morgante M, Hanafey M, Powell W. Microsatellites are preferentially associated with nonrepetitive DNA in plant genomes. Nat Genet. 2002;(30)2:194–200. 10.1038/ng822 11799393

[23] Toth G, Gaspari Z, Jurka J. Microsatellites in different eukaryotic genomes: survey and analysis. Genome Res. 2000;10:967–981. 10.1101/gr.10.7.967 10899146 PMC310925

[24] Mun J-H, Kim D-J, Choi H-K, Gish J, Debellé F, Mudge J, et al Distribution of Microsatellites in the Genome of *Medicago truncatula*: A Resource of Genetic Markers That Integrate Genetic and Physical Maps. Genetics. 2006;172(4):2541–2555. 10.1534/genetics.105.054791 16489220 PMC1456377

[25] Varshney RK, Graner A, Sorrells ME. Genic microsatellite markers in plants: features and applications. Trend Biotech. 2005;23:48–55. 10.1016/j.tibtech.2004.11.005 15629858

[26] Martin P, Makepeace K, Hill SA, Hood D W, Moxon ER. Microsatellite instability regulates transcription factor binding and gene expression. PNAS. 2005;102(10):3800–3804. 10.1073/pnas.0406805102 15728391 PMC553301

[27] Zhang L, Zuo K, Zhang F, Cao Y, Wang J, Zhang Y, et al Conservation of noncoding microsatellites in plants: implication for gene regulation. BMC Genomics. 2006;7:323 10.1186/1471-2164-7-323 17187690 PMC1781443

[28] Yu JK, Paik H, Choi JP, Han JH, Choe JK, Hur CG. Functional domain marker (FDM): an in silico demonstration in Solanaceae using simple sequence repeats (SSRs). Plant Mol. Biol. Rep. 2010;28:352–356. 10.1007/s11105-009-0154-8

[29] Kujur A, Bajaj D, Saxena MS, et al Functionally Relevant Microsatellite Markers From Chickpea Transcription Factor Genes for Efficient Genotyping Applications and Trait Association Mapping. DNA Research: An International Journal for Rapid Publication of Reports on Genes and Genomes. 2013;20(4):355–374. 10.1093/dnares/dst015 23633531 PMC3738162

[30] Liu W, Jia X, Liu Z, Zhang Z, Wang Y, Liu Z, Xie W. Development and characterization of transcription factor gene-derived microsatellite (TFGM) markers in *Medicago truncatula* and their transferability in leguminous and non-leguminous species. Molecules. 2015;20(5):8759–71. 10.3390/molecules20058759 25988608 PMC6272326

[31] Iquebal MA, S, Arora V, Verma N, Rai A, Kumar D. First whole genome based microsatellite DNA marker database of tomato for mapping and variety identification. BMC Plant Biology. 2013;13:197 10.1186/1471-2229-13-197

[32] Brouwer JR, Willemsen R, Oostra BA. Microsatellite repeat instability and neurological disease. BioEssays: news and reviews in molecular, cellular and developmental biology. 2009;31(1):71–83. 10.1002/bies.080122 PMC4321794 19154005

[33] Nelson DL, Orr HT, Warren ST. The Unstable Repeats—Three Evolving Faces of Neurological Disease. Neuron. 2013;77(5):825–843. 10.1016/j.neuron.2013.02.022 23473314 PMC3608403

[34] Golubov A, Yao Y, Maheshwari P, Bilichak A, Boyko A, Belzile F, Kovalchuk I. Microsatellite instability in Arabidopsis increases with plant development. Plant Physiol. 2010;154(3):1415–1427. 10.1104/pp.110.162933 20817752 PMC2971617

[35] Chen M, Tan Z, Zeng G, Peng J. Comprehensive Analysis of Simple Sequence Repeats in Pre-miRNAs. Mol Biol Evol. 2010;27:2227–2232. 10.1093/molbev/msq100 20395311

[36] Joy N, Asha S, Mallika V, Soniya EV. De novo Transcriptome Sequencing Reveals a Considerable Bias in the Incidence of Simple Sequence Repeats towards the Downstream of “Pre-miRNAs” of Black Pepper. Herrera-Estrella L, ed. PLoS ONE. 2013;8(3):e56694 10.1371/journal.pone.0056694 23469176 PMC3587635

[37] Cardle L, Ramsay L, Milbourne D, Macaulay M, Marshall D, Waugh R. Computational and experimental characterization of physically clustered simple sequence repeats in plants. Genetics. 2000;156:847–854. 11014830 10.1093/genetics/156.2.847 PMC1461288

[38] Mondal T, Ganie S. Identification and characterization of salt responsive miRNA-SSR markers in rice (*Oryza sativa*). Gene. 2014;535:204–209. 10.1016/j.gene.2013.11.033 24315823

